# Use of Mastectomy for Overdiagnosed Breast Cancer in the United States: Analysis of the SEER 9 Cancer Registries

**DOI:** 10.1155/2019/5072506

**Published:** 2019-01-22

**Authors:** C. Harding, F. Pompei, D. Burmistrov, R. Wilson

**Affiliations:** ^1^Data Scientist, Seattle, WA 98102, USA; ^2^Department of Physics, Harvard University, Cambridge, MA 02138, USA; ^3^Exergen Corp., Watertown, MA, USA; ^4^Worldpay, Lowell, MA, USA

## Abstract

**Aim:**

We investigated use of mastectomy as treatment for early breast cancer in the US and applied the resulting information to estimate the minimum and maximum rates at which mastectomy could plausibly be undergone by patients with overdiagnosed breast cancer. Little is currently known about overtreatments undergone by overdiagnosed patients.

**Methods:**

In the US, screening is often recommended at ages ≥40. The study population was women age ≥40 diagnosed with breast cancer in the US SEER 9 cancer registries during 2013 (n=26,017). We evaluated first-course surgical treatments and their associations with case characteristics. Additionally, a model was developed to estimate probability of mastectomy conditional on observed case characteristics. The model was then applied to evaluate possible rates of mastectomy in overdiagnosed patients. To obtain minimum and maximum plausible rates of this overtreatment, we respectively assumed the cases that were least and most likely to be treated by mastectomy had been overdiagnosed.

**Results:**

Of women diagnosed with breast cancer at age ≥40 in 2013, 33.8% received mastectomy. Mastectomy was common for most investigated breast cancer types, including for the early breast cancers among which overdiagnosis is thought to be most widespread: mastectomy was undergone in 26.4% of* in situ* and 28.0% of AJCC stage-I cases. These rates are substantively higher than in many European nations. The probability-based model indicated that between >0% and <18% of the study population could plausibly have undergone mastectomy for overdiagnosed cancer. This range reduced depending on the overdiagnosis rate, shrinking to >0% and <7% if 10% of breast cancers were overdiagnosed and >3% and <15% if 30% were overdiagnosed.

**Conclusions:**

Screening-associated overtreatment by mastectomy is considerably less common than overdiagnosis itself but should not be assumed to be negligible. Screening can prompt or prevent mastectomy, and the balance of this harm-benefit tradeoff is currently unclear.

## 1. Introduction

Here, we studied the use of mastectomy for early and overdiagnosed breast cancers. We specifically sought to evaluate how often overdiagnosed breast cancers were treated by mastectomy, which is a form of overtreatment. Although many studies have investigated the overdiagnosis of early breast cancer [1] or the use of mastectomy for early breast cancer, few have investigated both [2–9]. If sufficiently common, use of mastectomy for overdiagnosed breast cancer could be one of largest inadvertent harms in cancer treatment. We therefore thought it was worth studying. We conducted this evaluation using data from women diagnosed with breast cancer in 2013 in the Surveillance, Epidemiology, and End Results 9 US cancer registries.

The relationship between screening and mastectomy is complicated because screening can prevent mastectomies from being needed in some cases and cause mastectomies to be performed “unnecessarily” in other cases: If screening allows a harmful breast cancer to be detected at an earlier stage than would otherwise be possible, then use of mastectomy may be averted. On the other hand, if screening leads to overdiagnosis, then mastectomy may be performed “unnecessarily.” Because of this complication and because the overall rate of overdiagnosis is currently unknown and controversial [10, 11], it is not possible to calculate the exact amount of overtreatment by mastectomy that occurs after overdiagnosis. Instead, we have the more modest goal of determining minimum and maximum rates at which mastectomy could plausibly be undergone by overdiagnosed patients. (In other words, we aim to find lower and upper bounds on the rate of mastectomy in overdiagnosed patients.)

There is often an expectation that overdiagnosed patients rarely undergo mastectomy, based on the assumption that mastectomy is usually performed for aggressive-appearing cancers that are unlikely to be overdiagnosed. To date, however, this expectation has not been tested in the US. In placing bounds on how often overdiagnosed patients were treated by mastectomy, we sought to determine whether this expectation is justified.

## 2. Materials and Methods

### 2.1. Data Source

Data on 27,389 women diagnosed with* in situ* or invasive breast cancer in the year 2013 were obtained from Surveillance, Epidemiology, and End Results Cancer Registries (SEER) grouping 9, which includes the following regions: San Francisco-Oakland, Connecticut, the Detroit Metropolitan Area, Hawaii, Iowa, New Mexico, the Seattle Puget Sound Area, Utah, and the Atlanta Metropolitan Area. Of the 27,389 women, we excluded 1137 (4.2%) who were diagnosed with breast cancer at ages younger than 40, as well as an additional 235 (0.9%) for whom surgical treatment information was unavailable. The remaining 26,017 were included in our analyses, which amounts to about 9% of all women diagnosed with breast cancers in the US in 2013 [12].

The ages of included patients were limited to ≥40 because rates of mammography screening in the US are low before 40 and high afterward. For example, in the year 2010 Behavioral Risk Factor Surveillance System survey of the US population, receipt of at least 1 mammogram in the past 2 years was reported by approximately 8%, 9%, 46%, 77%, 81%, 83%, and 76% of women age 20, 30, 40, 50, 60, 70, and 80, respectively [13]. Screening participation increases suddenly at age 40 because several prominent US medical organizations recommend this as the preferred age to begin mammography screening [14]. Other studies of the US also report high mammography screening rates for ages ≥40, including older ages [15, 16].

The current study includes both screened women who were diagnosed with breast cancer and unscreened women who were diagnosed with breast cancer. This is because the SEER 9 registries do not record information on screening participation for individual patients. Nonetheless, the rate of screening participation is very high in the SEER 9 registry population as a whole. For example, data for 2008-2010 indicate that, of all women age ≥40 in the SEER 9 population, approximately 73% received at least 1 mammogram in the past 2 years [17]. We believe this high rate of screening participation makes the study population suitable for studying overdiagnosis and overtreatment, especially because the rate of screening participation was similar or lower in the screening arms of several of the randomized trials of mammography screening. For example, 74%, 68%, and 65% of the women assigned to screening arms of the Malmo I, UK Age, and New York HIP trials actually received their first screenings [18].

Some patients in our dataset had records for more than 1 breast cancer diagnosed in 2013 (*n*=1,049; 4.0%). For these patients, our analyses are of the surgical treatment and case characteristics in the registry records associated with the first of their year 2013 diagnoses. Before making this decision, we checked that only a negligible number of patients had different surgical treatments in the registry records associated with their first and later year 2013 diagnoses (*n*=68; 0.3%).

We analyzed SEER data on surgical treatments that were performed as part of first-course therapy [19, 20]. When the available case documentation did not provide enough information to determine whether therapy was first or later course, it was recorded in SEER as first course if given in the first year after diagnosis, and was considered to be later course if given in the second or later years after diagnosis [19].

All data used for this study are deidentified and publicly available from SEER using SEER*∗*Stat software.

### 2.2. Definitions

Total mastectomy was defined as simple mastectomy or modified radical mastectomy. Breast-conserving surgery was defined as lumpectomy, excisional biopsy, segmental/subtotal mastectomy, quadrantectomy, tylectomy, wedge resection, nipple resection, or partial mastectomy, not otherwise specified. SEER records the most extensive surgical procedure that was performed. In the overall cohort, mastectomy, breast-conserving surgery, other surgical therapies (including subcutaneous mastectomy), and no surgery of the primary site were performed in 33.8%, 56.4%, 1.3%, and 8.4% of included cases, respectively.

Breast cancer cases are defined as overdiagnosed if they were diagnosed because of screening, but if the cancer would not have been noticed or caused harm in the patient's lifetime in the absence of screening. Since overdiagnosed cancers do not require treatment, any treatment provided for them is regarded as overtreatment.

### 2.3. Estimation Approach

We sought to place bounds on how often mastectomy could plausibly be performed for overdiagnosed breast cancer. To obtain the bounds, three pieces of information were used: (A) a set of criteria that were used to rule out overdiagnosis in some cases, (B) an estimate of the proportion of breast cancer cases that are overdiagnosed, and (C) estimates of the probability of treatment by mastectomy for each case.

#### 2.3.1. Information A: Criteria Used to Rule out Overdiagnosis

We ruled out breast cancer cases from being overdiagnosed if they had any of the characteristics listed in Table 2. Because the characteristics reflect a behavior that is aggressive, advanced, and/or would quickly become clinically evident in the absence of screening, these presence of these characteristics indicates the breast cancer is highly unlikely to have been overdiagnosed.

For our bounds to be valid, we had to be especially careful that our criteria did not misclassify overdiagnoses as nonoverdiagnoses. As a consequence, some of the criteria in Table 2 may appear overly conservative. For example, because 2.0-3.9 cm tumors could be overdiagnoses in rare cases, we did not exclude them. Had they been excluded, our bounds might have been rendered invalid, especially since mastectomy becomes more common at larger sizes. Similarly, we did not rule out cases with 1 positive lymph node because they could occasionally be overdiagnoses with a false-positive lymph node, and mastectomy might be especially common for these cases. (False-positive lymph node biopsy findings have been reported [21–23], though the false-positive rate appears to be unknown.)

We have tried to be suitably conservative when selecting these criteria, but we realize that some will debate our choices. To address this, we conducted supplementary analyses in which we tried alternative criteria and examined how estimates of overtreatment by mastectomy were affected. For example, we tried ruling out overdiagnosis for cases with ≥1 positive lymph node and/or tumor sizes of ≥3.0 cm, and found that our bounds on use of mastectomy for overdiagnosed cancer changed by only a couple of percentage points. Accordingly, our judgments of how many lymph nodes and what tumor sizes fully rule out overdiagnosis did not have large consequences for our results. More details can be found in the supporting information (Table S2 and Figure S1).

#### 2.3.2. Information B: Estimates of the Proportion of Breast Cancers That Are Overdiagnosed

The amount of overdiagnosis that is occurring is not clear and, in the prior literature, estimates of overdiagnosis rates have ranged widely from <1% to >50%, changing greatly depending on study designs, settings, and measures of overdiagnosis [10, 24–28]. To account for this variation, we performed our analyses several times, using different estimated values for the proportion of breast cancers in the study population that were overdiagnosed. The range of investigated values was 0% to 37%. We chose this range based on the following considerations: In the SEER 9 cancer registries, mammography screening was rare during and before 1980. Since then, both screening rates and breast cancer incidence have increased [29]. Assuming that the incidence of nonoverdiagnosed breast cancer incidence has either been constant or increasing over 1980-2013, and that mammography screening is responsible for almost all overdiagnoses of breast cancer, then the rate of nonoverdiagnosed breast cancer cannot be substantively lower than the incidence rate observed in 1980, and the rate of overdiagnosis cannot be substantively higher than the overall increase in breast cancer incidence from 1980 to 2013. So, whatever it is, the true amount of overdiagnosis lies between these two values. Among women age ≥40 in the SEER 9 cancer registries, the age-standardized incidence of breast cancer was 230.1 per 100,000 in 1980 and 364.6 per 100,000 in 2013. Therefore, under the noted assumptions, at least 0% and at most 37% of breast cancers in the study population could be overdiagnosed (37% = 1 − 230.1/364.6).

#### 2.3.3. Information C: Estimates of the Probability of Treatment by Mastectomy

We used a regression analysis to estimate the probability of treatment with mastectomy according to the recorded characteristics of the cases in the study population at diagnosis. Thirty-three characteristics were included in our analysis, including various patient, disease, and regional attributes (Table S1).

If we had used only a couple of characteristics—say stage and grade—then determining the probability of mastectomy would not require regression. Instead, we would simply calculate the proportion of cases treated by mastectomy for each unique combination of stage and grade. (In other words, we would create a cross-table.) However, as the number of characteristic increases, the number of unique combinations that need to be considered becomes huge, making estimates of the proportion of cases treated by mastectomy unstable. To address this sparse-data problem, we used regression modeling to estimate the probabilities of treatment by mastectomy, instead of calculating these values directly in cross-tables. We performed the regression using a random forest model. This is a common, basic method from the machine learning literature that was selected because it offers reliable performance, is resilient to the curse of dimensionality, and does not generally overfit [30–32].

Using the randomforestSRC package [31, 33], a random forest model was trained with 2500 trees, the square root of the total number of variables as the number of variables tried per node split, Gini index splitting, a leaf size of 1, and a maximum of 25 random splits for multivalue variables. These hyperparameters were not tuned. The random forest was fit to cases diagnosed in 2013 (training set) and tested on cases diagnosed in 2012 (test set). For the year 2013 probabilities of mastectomy analyzed in this article, we used out-of-bag estimates to avoid overfitting. The calibration of the random forest was good for both the training and test set (Figure S2). In regard to accuracy and discriminative performance, Breir score values were 0.176 for 2012 and 0.175 for 2013, and c-statistic values (areas under the receiver operating characteristic curves) were 0.745 for 2012 and 0.742 for 2013. Because the bounds obtained in our analysis are dependent on the discriminative performance of the fitted model, we also performed sensitivity analyses in which investigated whether performance was substantially changed by fitting the model on half (random sample of 2013) and twice (years 2012 and 2013 together) as many records, and by using half and twice as many trees. Calibrations curves, Breier scores, and c-statistics values were similar to those reported above, as were the lower and upper bounds on the frequency of mastectomy for overdiagnosed cancer. These and all other statistical analyses were conducted in R (The R Foundation for Statistical Computing; Vienna, Austria).

#### 2.3.4. Estimating Overtreatment by Mastectomy

We obtained bounds on the frequency at which mastectomy is performed for overdiagnosed cancer by applying Information A, B, and C. The following steps were used: First, we excluded all cases that had characteristics ruling out overdiagnosis (applying Information A). Second, we considered that each remaining case belonged to one of two groups, the overdiagnosed group or the nonoverdiagnosed group, but that the membership of these groups was not observable. We assumed that the overdiagnosed group had a specific size (applying Information B). Third, we analyzed how the probability of treatment by mastectomy varied according to characteristics at diagnosis (applying Information C). To obtain a minimum plausible estimate (lower bound) of how often mastectomy was performed for overdiagnosed cancer, we filled up the overdiagnosed group with the cases that had the least probabilities of treatment by mastectomy. On the other hand, to obtain a maximum plausible estimate (upper bound), we filled up the overdiagnosed group with the cases that had the greatest probabilities of treatment by mastectomy.

For example, suppose that we rule out the cases that cannot be overdiagnoses and are left with 75% of the original study cohort. Suppose also that 30% of the entire cohort are overdiagnoses. Then, simple calculation shows that 40% of the remaining cases are overdiagnoses [40% = 30% / 75%]. We do not know which breast cancer cases belong to the 40% that are overdiagnoses, and this prevents us from calculating exactly how common it is for overdiagnosed cancers to be treated by mastectomy. However, we can still make progress based on a key observation: No one is able to identify overdiagnosed cases; therefore, the probability of treatment by mastectomy is the same for overdiagnosed and nonoverdiagnosed cases that share the same observed characteristics. So, we reason that the actual frequency of mastectomy-treated overdiagnoses cannot reasonably be less than it would be if the overdiagnosed cases were the cases that had characteristics associated with the lowest probability of treatment by mastectomy. Similarly, the actual frequency of mastectomy-treated overdiagnoses cannot reasonably be greater than it would be if the overdiagnosed cases were the cases that had characteristics associated with the greatest probability of treatment by mastectomy. In this way, we obtain minimum and maximum plausible estimates of the frequency at which mastectomy is performed for overdiagnosed breast cancer.

In more statistical detail, our approach is as follows: After excluding the cases that cannot be overdiagnoses (Information A), we are left with* n* cases, some of which are overdiagnosed and others of which are nonoverdiagnosed. Denote by* X* the 33 characteristics included in our regression analyses (Information C), and let the values of these characteristics for case* i* be* x*_*i*_. Further, let* M *denote that mastectomy was performed and* V* denote that overdiagnosis occurred. We explain the method of obtaining bounds in the large-sample limit, which is a good approximation for the analysis in this paper because of the very large-sample size.

We are interested in estimating the proportion of the* n* cases in which mastectomy was performed for overdiagnosed cancer. This is,

(1) PrM,V=∑xiPrM ∣ V,X=xi·PrV ∣ X=xi·PrX=xi

Currently, no one can identify cases that have been overdiagnosed. (Indeed, if overdiagnosed cases could be identified, they would not be treated, and there would be no need for our study.) For this reason, we make our key assumption: Conditional on the observed characteristics of a case at diagnosis, the probability of mastectomy would not be different if the case was overdiagnosed cancer or if it was nonoverdiagnosed cancer. The overdiagnosed and nonoverdiagnosed cases are then exchangeable conditional on observed characteristics.

(2) PrM ∣ V,X=xi=PrM ∣ ¬V,X=xi=PrM ∣ X=xi

Plugging these results into our expression for the proportion of cases with mastectomy after overdiagnosis (Expr. (1)), we have

(3) PrM,V=∑xiPrM ∣ X=xi·PrV ∣ X=xi·PrX=xi

In this expression, Pr(*M*∣*X* = *x*_*i*_) is estimated using a regression model (Information C), while Pr(*X* = *x*_*i*_) is estimated by the proportion of all cases in the study cohort with *X* = *x*_*i*_. Only Pr(*V*∣*X* = *x*_*i*_), the probability of overdiagnosis conditional on the observed characteristics, is unknown.

If we assume that the proportion of the* n *cases that are overdiagnosed takes a known value, say* q* (Information B), then this restricts the values that Pr(*V*∣*X* = *x*_*i*_) can take. By distributing the allowed values of Pr(*V*∣*X* = *x*_*i*_) in such a way that maximizes the value of Expr. (3), we obtain an upper bound on the frequency of mastectomy-treated overdiagnoses. Similarly, by distributing the allowed values of Pr(*V*∣*X* = *x*_*i*_) to minimize Expr. (3), we obtain a lower bound.

In practice, the upper bound is obtained simply by assigning a value of 1 to Pr(*V*∣*X* = *x*_*i*_) for the proportion* q *of cases for which Pr(*M*∣*X* = *x*_*i*_) is largest, and assigning a value of 0 otherwise. Similarly, lower bound is obtained by assigning 1 to Pr(*V*∣*X* = *x*_*i*_) for the proportion* q *of cases for which Pr(*M*∣*X* = *x*_*i*_) is smallest, and assigning 0 otherwise. In this way, we obtain bounds on the proportion of breast cancers cases in the study population that were overdiagnosed and overtreated by mastectomy.

Appendix S1 provides additional detail, including discussion of the key independence/exchangeability assumption and explanation of how our approach relates to other methods, such as propensity scores and regression standardization.

### 2.4. Sensitivity Analysis for Omitted Variables

Though the main analysis of this study includes adjustment for 33 variables, several variables relevant to use of mastectomy were not recorded in our data source and therefore could not be adjusted for. For example, the data source did not record most cancer symptoms, breast cancer-related mutations (e.g.,* BRCA* mutations), family histories, screening histories, or whether mastectomy became necessary following breast-conserving surgery (e.g., due to recurrence or incomplete resection). Additionally, the data source often had missing values, and missingness could be informative of surgical choices in some cases. For these reasons, we performed an additional analysis in which we investigated the sensitivity of our results to any omitted variables and missing values that are relevant to use of mastectomy. A full explanation of the method is given in Appendix S1. In brief, the sensitivity analysis assumes that the predictions of mastectomy use are not systematically biased, but that omitted variables and missing values could increase their variance. The analysis is governed by a sensitivity parameter, which is the largest odds ratio (OR) by which omitted variables and missing data can change the probabilities of mastectomy from their estimated values. We used ORs that ranged from 1 to 25 to evaluate the maximum extent to which our results could be changed by omitted variables and missing data.

### 2.5. Separate Analysis of* Ductal Carcinoma In Situ*

In a supplementary analysis, we repeated our evaluation of overtreatment by mastectomy for women diagnosed with ductal carcinoma* in situ* (DCIS), specifically. The supplemental analysis proceeded identically to the main analysis, with two exceptions: First, instead of using the rule-out criteria shown in Table 2, we ruled out all cases that were not DCIS. DCIS was defined as* in situ* breast cancer with ICD-O-3 code 8201, 8230, 8500-8507, or 8523 [34]. Second, the range of possible overdiagnosis rates was changed from 0-37% to 0-90% for DCIS cases, with the maximum of this range chosen based on the observation that DCIS incidence increased from 6.5 to 66.2 per 100,000 from 1980 to 2013 among women age ≥40 in SEER 9 (90% = 1 – 6.5/66.2; the calculation is analogous to that reported for overall breast cancer in Section 2.3).

### 2.6. Interpretation of Bounds

Our bounds estimate the minimum and maximum plausible percentages of the study population who underwent mastectomy for overdiagnosed breast cancer. The study population is all women in the SEER 9 registries who were diagnosed with breast cancer (screen-detected or clinically detected) at age ≥40 in 2013.

When interpreting the bounds given in our results and figures, it is important to remember that they do not provide any information about the location of the true value within the bounds. They merely show the values that are plausible. For example, if our methods show 3%-15% of cases are overdiagnoses treated by mastectomy, then this does not provide any information about whether the true value is near the middle of this range, 9%, or nearer the edges. Further, the bounds do not tell us about the rate of overtreatment in years or areas other than those included in the study population. For example, our findings are for 2013, and the rate of mastectomy-treated overdiagnoses is likely somewhat different today. Finally, although the ranges tell us about the rate of mastectomy-treated overdiagnoses in the study population as a whole, they do not provide any information about the probability of overtreatment by mastectomy for individual patients. If we are considering an individual patient, then the probability that she received mastectomy for overdiagnosed cancer can be lower or higher than the range, depending on the characteristics of her case.

When reporting bounds, we rounded the percentages outwards to be conservative. For example, a bound of 5.6%-12.4% was rounded to 5%-13%.

## 3. Results

### 3.1. Overall Use of Mastectomy

Table 1 summarizes the characteristics of the study population: women diagnosed with breast cancer at age ≥40 in 2013 in the SEER 9 cancer registries. Overall, 33.8% of the 26,017 included patients received treatment by mastectomy. Larger tumor sizes and younger patient ages were associated with progressively higher rates of mastectomy (p < 0.0001 for each trend; *χ*^2^ test for trend). However, inspecting the percentage values shows that mastectomy was common for all categories investigated in the Table, including for all tumor sizes and all ages.

Use of mastectomy was also common for* in situ* breast cancers: overall, 26.4% of patients with* in situ *breast cancers were treated with mastectomy. Among patients with* in situ* cancer, higher mastectomy rates were associated with larger tumors (19.9%, 25.8%, 31.7%, 40.0%, 43.4%, and 61.8% for 0.0-0.9, 1.0-1.9, 2.0-2.9, 3.0-3.9, 4.0-4.9, and 5.0 cm+, respectively), younger ages (35.1%, 26.5%, 21.3%, and 17.6% for ages 40-49, 50-64, 65-84, and 85+, respectively), and higher grades (18.1%, 25.7%, and 34.2% for grade I, II, and III+, respectively).

### 3.2. Use of Mastectomy for Overdiagnosed Breast Cancer

We estimated use of mastectomy for overdiagnosed breast cancer using a multistep process. The first step of the process was to exclude women with case characteristics that ruled them out from being overdiagnosed (Table 2; Methods). After the exclusion, 20,220 women (77.0%) remained. The characteristics of their cases are compared with rates of mastectomy in Table 3.

Excluding cases that were inconsistent with overdiagnosis reduced the rate of mastectomy slightly, from 33.8% to 28.8%. Larger tumor sizes and younger patient ages continued to be associated with higher rates of mastectomy (p < 0.0001 for each trend; *χ*^2^ test for trend). Rates of mastectomy remained relatively high for all investigated categories of breast cancer, including* in situ* cancers. The presence of high rates of mastectomy for all investigated categories suggests that mastectomy was also common for overdiagnosed cases.

Applying the remaining steps of the estimation process (see Methods), we found that at most 18% of women diagnosed with breast cancer at age ≥40 in 2013 had overdiagnosed cancers that were treated by mastectomy.

**Figure 1:**
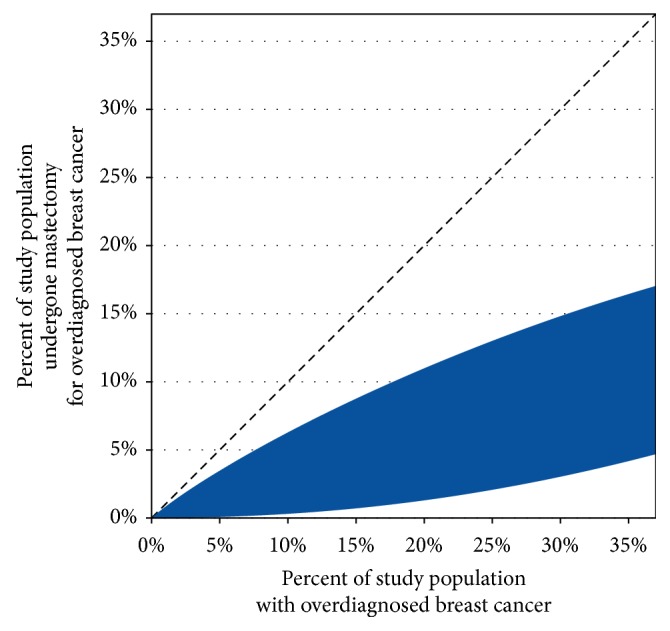
Main analysis of overtreatment by mastectomy in the study population, which consists of all women diagnosed with breast cancer at age ≥40 in 2013 in the SEER 9 cancer registries. The percentage of patients with breast cancer who undergo mastectomy for overdiagnosed cancer is unknown. However, our main analysis determines the range of possible values that are consistent with the known characteristics of breast cancer cases in the study population. Specifically, in the above figure, the x axis shows the percentage of the study population who were overdiagnosed, the y axis shows the percentage of the study population who underwent mastectomy for overdiagnosed cancer, and the shaded region shows the range of values that are consistent with the known characteristics of breast cancer cases. For example, if 20% of the study population was overdiagnosed then, based on the known characteristics of the breast cancer cases, we can conclude that somewhere between 1% and 11% of the study population received mastectomy for overdiagnosed cancer. As another example, if 0% of the study population was overdiagnosed, then 0% received mastectomy for overdiagnosed cancer. Additionally, if 37% was overdiagnosed, then somewhere between 5% and 18% received mastectomy for overdiagnosed cancer. (The figure shows a range of possible values for percentage overdiagnosed because there is little consensus regarding the true number, with potential values extending from near 0% to 37%.) The analysis includes both invasive and* in situ* breast cancers.

Since the rate of overdiagnosis is currently unclear, the influence of the rate of overdiagnosis on our estimates was also evaluated. Supposing 1%, 5%, 10%, 20%, or 30% of breast cancers in the study population were overdiagnosed, we found that 0%-1%, 0%-4%, 0%-7%, 1%-11%, or 3%-15% of breast cancers in the study population were overdiagnoses that had been treated by mastectomy. The complete relationship between overdiagnosis rates and mastectomy use for overdiagnosed cancer is shown in Figure 1.

The results of our analysis can be combined with previously published estimates of overdiagnosis rates. For example, in a previous study, we estimated that 31% (95% CI: 28-34%) of breast cancers were overdiagnosed in the US during 1996-2009 [35], which amounts to 33% of breast cancers in women age ≥40. Additionally, Bleyer and Welch estimated that 31% of breast cancers in women age ≥40 were overdiagnosed in the US during 2008 [29]. Using either the 33% value or the 31% value, reference to the figure shows that 3%-16% of breast cancers are both overdiagnosed and treated with mastectomy. (The same range is obtained for both 33% and 31% because these values are so similar.) However, if almost no breast cancers are overdiagnosed, as has been argued for example by Feig [36], then naturally close to 0% of breast cancers are both overdiagnosed and treated with mastectomy.

### 3.3. Sensitivity Analysis for Omitted Variables

Our main analysis (i.e., Figure 1) relies on estimates of the probability of mastectomy that were adjusted for 33 patient and case characteristics recorded in the cancer registry data. However, data values were sometimes missing and some unrecorded variables could be relevant to the selection of mastectomy as the patient's treatment. We performed a sensitivity analysis to address the possibility of bias from this omitted information.

**Figure 2:**
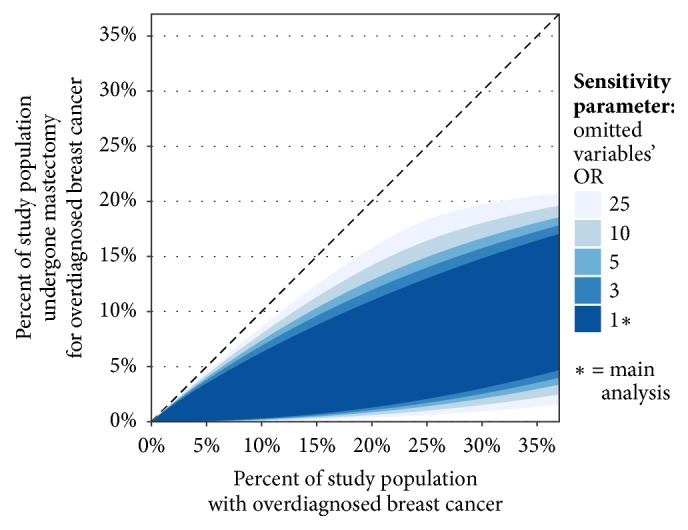
Sensitivity analysis showing the robustness of the study findings to omitted variable bias. We investigated the sensitivity of our main analysis to the existence of omitted variables that are associated with use of mastectomy. The sensitivity analysis is governed by a sensitivity parameter, which is the largest odds ratio (OR) by which the omitted variables can change probabilities of mastectomy from the values estimated in the main analysis. (In the main analysis, probabilities of mastectomy were estimated using 33 patient and case variables. However, some relevant variables were unavailable.) The figure presents results for several sensitivity parameter values, ranging from OR=1 to OR=25. Results for OR=1 are equivalent to the main analysis. On the other hand, OR=5 is equivalent to the omission of a very important determinant of mastectomy; among all the variables in Table 3, the largest OR was 4.9. Accordingly, the results in the figure show that even omitted variables with large ORs produce little change from the main analysis. Therefore, the study findings are largely robust to omitted variables. Appendix S1 includes a full description of the sensitivity analysis and definition of the sensitivity parameter.

Results of the sensitivity analysis are shown in Figure 2. The analysis is governed by a sensitivity parameter, which is the largest OR by which omitted variables and missing data can change probabilities of mastectomy from the values estimated in the main analysis. ORs ranging from 1 to 25 were investigated. For context, the largest mastectomy OR for the variables reported in Table 3 is 4.9 (95% CI: 4.3-5.6; univariate analysis of regional nodes positive, 1 positive node versus unknown nodal status). Based on the results shown in Figure 2, a single omitted variable that had a similarly large association with mastectomy could at worst affect our results mildly (OR ≈ 5), and two such omitted variables (largest possible combined OR ≈ 25) could at worst affect our results moderately. In summary, the results of the main analysis are largely robust to bias from omitted variables and missing data. This is both a feature of the specific statistical approach that we employed (Appendix S1), and a byproduct of the relatively wide range of the bounds.

### 3.4. Sensitivity Analyses for Rule-Out Criteria

As an additional sensitivity check, we applied alternative sets of criteria to rule out overdiagnosis (Table S2). Minimum plausible estimates (lower bounds) on the rate of mastectomy-treated overdiagnosis were largely independent of the choice of criteria. Maximum plausible estimates (upper bounds) varied by a few percentage points depending on the criteria that were used (Figure S1).

### 3.5. Separate Analysis of Mastectomy Use for Overdiagnosed DCIS

In a supplementary analysis, we repeated our evaluation of mastectomy use in overdiagnosed cases for women diagnosed with DCIS, specifically. The study cohort included 4666 women diagnosed with DCIS, which amounts to 85.6% of the women diagnosed with* in situ *breast cancer. Mastectomy was undergone by 27.4% of the women with DCIS. Our statistical methods indicated that at most 27% of the women with DCIS could have been overdiagnosed and subsequently treated by mastectomy (Figure S3).

As previously, our results depend on the proportion of women who were overdiagnosed. For DCIS, rough estimates of this proportion can be obtained through follow-up of cases in which the tumor was misdiagnosed as benign and therefore treated minimally—with biopsy only. In a review of such cases, Erbas et al. found that only 14-53% proceeded to invasive breast cancer during follow-up of 10-15 years [37]. This suggests that somewhere near 47-86% of DCIS tumors are practically nonprogressive and overdiagnosed (47% = 100% – 53%; 86% = 100% – 14%). If the 47% value is correct, then our analysis shows that 2-25% of women with DCIS were overdiagnosed and treated with mastectomy. On the other hand, if the 86% value is correct, this range changes to 16-27%.

## 4. Discussion

### 4.1. Main Findings

In this study of surgical treatment in a large US cancer registry, mastectomy was undergone by 33.8% of women diagnosed with breast cancer at age ≥40. Mastectomy was relatively common for breast cancer cases of all investigated stages, sizes, lymph node statuses, grades, molecular types, and ages of diagnosis (range of mastectomy rates, 16.9%-62.7%; Table 1). Notably, mastectomy was common for the early breast cancers among which overdiagnosis is thought to be most widespread: mastectomy was undergone in 27.4% of DCIS cases and 28.0% of AJCC stage-I cases.

The amount of overdiagnosis associated with screening is controversial. Prior estimates of overdiagnosis rates range from <1% to >50%, depending on the assumptions, populations, and measures of overdiagnosis used by researchers [10, 24–28]. For the present article, we sought to put aside the controversial question of how much overdiagnosis is occurring, and instead aimed to clarify the relationship between overdiagnosis and overtreatment by mastectomy.

The study population was women age ≥40 who were diagnosed with breast cancer in 2013 in the SEER 9 cancer registries. The analysis included both* in situ* and invasive breast cancers and both screen- and clinically detected breast cancers. We determined that at most 18% of the study population underwent mastectomy for overdiagnosed breast cancer. Because the SEER 9 cancer registries include 9.6% of the US population and are broadly representative of the US [38], it is possible to scale our results up proportionally in order to obtain a rough estimate of the maximum plausible frequency at which mastectomy is performed for overdiagnosed breast cancer in the US as a whole. Doing so, we find the following: Of the approximately 297,000 US women who were diagnosed with breast cancer at any age during 2013 [12], a maximum of 47,000 underwent mastectomy for overdiagnosed breast cancer. That this value is so high is largely attributable to the presence of relatively high mastectomy rates for all investigated categories of breast cancer, including for early disease stages (Table 1).

In contrast to these high values, the minimum plausible rate of mastectomy-treated overdiagnosis that is indicated by our statistical methods is 0. Nonetheless, the statistical methods account for only some of what is known about breast cancer treatment, and commonsense reasoning suggests that breast cancer screening will lead to mastectomy being performed for at least a small proportion of overdiagnosed tumors, since mastectomy is undergone by a large proportion (27.4%) of women diagnosed with DCIS (see also [39]), and because it is generally agreed that at least some DCIS cases are overdiagnosed.

In summary, when we used formal methods to investigate how often mastectomy may be performed for overdiagnosed breast cancer, we found that the available data rule out neither low nor high rates of this overtreatment (Figure 1). This is concerning because the overtreatment of overdiagnosed cases by mastectomy has the potential to be one of the larger medical harms in oncology. Yet, even when studied in detail, it is currently difficult to determine whether the harm is common or nearly nonexistent.

Many factors contribute to the decision to undergo mastectomy, including the patient's own preferences and personal assessments of benefits and risks, as well as regional differences in practice patterns [40]. Because many factors are involved, it is arguable whether the selection and use of mastectomy for overdiagnosed tumors should be attributed to screening alone. Without screening, however, none of the overdiagnosed tumors would have been detected in the first place and, therefore, overtreatments of these tumors would be entirely avoided.

### 4.2. Previous Studies of Mastectomy Rates in the US

The high rates of mastectomy that we have observed in the US data are supported by prior research. For example, in a large US study of breast cancers diagnosed in 1998-2011, Kummerow et al. found that mastectomy was undergone by 35.5% of women with T0-2, N0-2, M0 breast cancers and 29.3% of women with* in situ* breast cancers [41]. Further, in a large US study of 2007-2011, Ward et al. found that mastectomy was undergone by 27% of women diagnosed with* in situ *breast cancer [34]. However, neither Kummerow et al. nor Ward et al. mentioned the implications of these high rates of mastectomy for overtreatment of overdiagnosed breast cancer, or used the word “overdiagnosis” at all. As seen from the results of the current study, the high rates of mastectomy for early breast cancers suggest that many overdiagnosed breast cancers will also be treated by mastectomy.

### 4.3. Previous Studies of Mastectomy Use for Overdiagnosed Cancer

Several previous studies have also investigated treatment of overdiagnosed breast cancer by mastectomy. In the Cochrane review of mammography screening [9], a meta-analysis of 5 randomized trials (2 from Canada and 3 from Sweden) showed that 20% more mastectomies and 30% more breast operations overall (mastectomies plus lumpectomies) were performed in women who had been randomized to mammography screening than in control groups (relative rates: mastectomy, 1.20, 95% CI 1.11–1.30; overall breast operations, 1.35, 95% CI 1.26–1.44). As noted by the review's authors [9], this finding is consistent with substantial overtreatment by mastectomy. However, the meta-analysis was limited to surgical treatments performed in the 1970s–1990s, when mastectomy was more common and breast-conserving surgery was less available than is the case today.

In observational studies, the introduction of mammography screening in Norway and Denmark was observed to be accompanied by increases in use of mastectomy, and these increases were attributed in part to overtreatment of overdiagnosed patients [2, 6]. Further, a study of DCIS diagnosed in England found that screening was associated with increased mastectomy, although invasive cancers were not investigated [4]. On the other hand, in two studies set in Italy, the introduction of screening was accompanied by reduced use of mastectomy, which the authors attributed to benefits of screening [5, 7]. Additionally, in a simulation-based study of women diagnosed with breast cancer at ages 50–74 in Isère, France, Seigneurin et al. concluded that only 1.4% (95% CI 0.2–2.6%) of screen-detected breast cancers were overdiagnoses that were treated by mastectomy [8].

Most recently, in an observational single-center analysis of 791 Australian women with stage 0-3A invasive breast cancer, Elder et al. [42] found that mastectomy, axillary dissection, adjuvant chemotherapy, and postmastectomy radiotherapy were all less likely to be recommended by physicians for patients who were active screeners, as compared to patients without recent screening. The difference between mastectomy receipt among actively screened patients versus not-recently screened patients was especially striking (17% versus 33% undergoing mastectomy, respectively), and Elder et al. continued to find that screening participation was associated with reductions in recommended treatment intensity after applying a correction for 22% overdiagnosis among the breast cancer patients. However, it is not clear if this correction was sufficient given that studies have reported substantially higher overdiagnosis percentages in Australia (e.g., 30-42% of invasive cancers overdiagnosed among women age 50-69 [43]). Additionally, it is not clear how well results from the single hospital studied by Elder et al. generalize to other locations.

Most of the previous studies were conducted in Europe. Compared with many European nations, we expect rates of mastectomy-treated overdiagnoses to be higher in the US because US women are recommended to begin screening earlier (often at age ≥40) and receive it more frequently (often yearly), both of which are expected to lead to more overdiagnosis and, consequently, more overtreatment. Furthermore, the high rates of mastectomy in the US will contribute to overtreatment. Garcia-Etienne et al. found that mastectomy rates were decreasing in Europe for early-stage breast cancers (stage 0–II, excluding pT3) [44, 45], reaching 13.1% in 2010. By comparison, the mastectomy rate was more than twice as great for comparable patients in our study, and research by Kummerow et al. suggests that the mastectomy rate has been increasing in the US [41], perhaps as a consequence of changing patient and physician concerns, or the increasing performance of mastectomy as an outpatient procedure. In notable contrast to the increasing use of mastectomy in the US, several recent observational studies have reported that patients treated with breast-conserving surgery have superior survival to those treated with mastectomy, even after controlling for many potential confounders [46].

### 4.4. Prevention of Mastectomy by Screening

Besides being a cause of overtreatment, screening can also prevent mastectomy from being needed by catching harmful tumors earlier and thereby allowing breast-conserving surgery to be performed instead. The present study did not focus on this benefit of screening, but some of our findings are still relevant. In particular, we found that mastectomy rates are reduced with reducing stage and tumor size, but increased with lowering age (Table 1). In terms of screening's effects on use of mastectomy in US, this suggests that screening may reduce the use of mastectomy for nonoverdiagnosed breast cancers, but that some of the benefits accrued from detecting these cancers at earlier stages and smaller sizes may be counteracted by their detection at younger ages. This would cut into any benefits that may derive from starting screening at younger ages and is therefore relevant to the current debate over whether screening mammography should be started at 40 or 50 years of age.

Previous studies have also investigated the prevention of mastectomy by screening. As mentioned above, a meta-analysis of 5 randomized trials found that the mastectomy rate was 20% greater in screening arms than control arms, indicating that screening was preventing substantially fewer mastectomies than it was prompting [9]. However, Elder et al.'s Australian study showed that the percentage of breast cancer patients undergoing mastectomy was markedly lower among active screeners than among those without recent screening, suggesting the opposite [42]. Finally, two large-scale ecological analyses showed that the incidence of breast cancer treated by mastectomy was broadly similar in high- and low-screening regions of the US [35, 47], suggesting either that the harm of overtreatment by mastectomy and the benefit of prevented mastectomy are both uncommon, or that they are of approximately equal size. Since the relevant studies conflict and all have substantial limitations, further research is needed to determine how often screening allows mastectomy to be replaced with breast-conserving surgery.

### 4.5. Other Forms of Overtreatment for Overdiagnosed Breast Cancer

The present study did not assess burdens imposed by overtreatments by breast-conserving surgery, reoperation [48], radiotherapy, chemotherapy [49], hormone therapy, and other therapies, which can be substantial. The risks of chemotherapy, radiotherapy, and other therapies for overdiagnosed patients are concerning given the long-term adverse effects to heart health and increased rates of deaths from heart disease among breast cancer survivors [50–52].

Comparing breast-conserving surgeries and mastectomies is a natural division point, particularly as part of the purpose of breast-conserving surgery is to be less life-changing than mastectomy. However, other division points might be investigated in future research, such as in-patient versus outpatient procedures, or breast-conserving surgeries without axillary dissection versus other surgeries. Given the increasing use of contralateral prophylactic mastectomy for early-stage breast cancer in the US [53, 54], including for DCIS [34, 55], the rate at which overdiagnosis results in bilateral mastectomy is also worth investigation. Moreover, it would be beneficial to expand the analysis to evaluate the use of radical mastectomy in cases that are not overdiagnosed, but for which breast-conserving surgery is expected to be sufficient, for example based on long-term follow-up of randomized trials of surgical treatment that predate widespread mammography screening [56].

### 4.6. Use of Mastectomy at Younger Ages

In this study, we also observed that use of mastectomy is especially common among women diagnosed with* in situ *breast cancer at younger ages. For example, 37.6% of 40–44-year-old women diagnosed with* in situ* breast cancer in 2013 underwent mastectomy as part of first-line treatment, whereas 22.8% of those aged 70–74 years did. In addition to the high rate of mastectomy at younger ages, there is also a trend of increasing use of mastectomy in the US overall. In a study of more than 1.2 million breast cancer patients in the National Cancer Data Base, Kummerow et al. found that women who are eligible for breast-conserving surgery have increasingly been undergoing mastectomy instead and that increases in use of mastectomy have been especially great for* in situ* cases, node-negative cases, and cases with small tumors [41]. Further study of these trends is warranted to determine the causes.

### 4.7. Study Limitations

There are several limitations to our study. In the sensitivity analyses, we have evaluated limitations related to the choice of criteria that were used to rule out overdiagnosis (Figure S1), as well as limitations related to omitted variables. The issue of omitted variables deserves special attention because several potentially relevant variables were unavailable in the cancer registry data source, such as mode of cancer detection. However, a feature of our methods is that they are robust to omitted variables, as shown in Figure 2. This robustness becomes more evident if one considers that the main results (Figure 1) already account for the portions of the associations between omitted variables and mastectomy that occur via correlation with the 33 available variables, since these available variables are already included in the regression that is used to estimate mastectomy probability. So, the sensitivity analysis is only needed to address any residual association that remains between omitted variables and mastectomy, after controlling for the available variables in the regression.

For example, consider mode of cancer detection (screening versus symptomatic). This omitted variable could have a strong association with mastectomy use, but we expect that most of its association would be attributable to the correlations that exist between mode of detection and other variables that affect selection of mastectomy more directly, like tumor size, lymph node status, and stage. These variables are already included in our main analysis, along with 30 more (Table S1). The sensitivity analysis only needs to address whatever residual association remains between mode of detection and mastectomy, after controlling for all the associations that tumor size, lymph node status, stage, and the other included variables have with mastectomy. We expect this residual association to be comparatively small, and addressed fully within the sensitivity analysis.

Another limitation is that a better-performing model or larger sample sizes would result in more discriminative estimates of mastectomy probability which, if the improvement were large enough, could affect the bounds. As noted in the Methods, our results were not substantially affected by halving or doubling the sample size, or by using forests with half or twice as many trees. Additionally, since the sensitivity analysis in Figure 2 maintains calibration while it increases discrimination, it also serves as a check on how much our results could change if we used a better-performing model or increased sample sizes. However, use of substantially different models may produce larger changes in the results.

An additional limitation is that the analysis of 1980-2013 trends assumes there was negligible overdiagnosis of breast cancer in 1980. However, screening by physical examination was common at that time and, if it led to overdiagnosis, then the overdiagnosis rate in 2013 could conceivably be higher than 37%, meaning that the upper bound on overtreatment by mastectomy would increase above 18% (Figure 1). The 18% upper bound would also increase if the incidence of nonoverdiagnosed breast cancer decreased during 1980-2013. Another limitation is that some overdiagnoses among those with short life expectancy may be classified as nonoverdiagnosed using our criteria (Table 2), since cases with advanced features can be overdiagnosed in this population [10]. This could also result in some underestimation of the rate at which mastectomy is provided for overdiagnosed cancer, especially among elderly patients. Finally, although SEER 9 is broadly representative of the US, it over-represents some population groups (such as city residents) and will not be exactly reflective of breast cancer or surgical treatment rates in the US as a whole.

When considering the value of our method, it should be judged in the context of available alternatives. The most common alternative method in the literature is to examine how population-level trends in mastectomy rates varied as screening was introduced. This method has been applied to European counties [2, 4–6], but is subject to its own limitations, and is in any case difficult or impossible to apply in the US because the advent of screening in the US was gradual, and corresponded with unrelated trends in practice that led to increasing rates of breast-conserving surgery. Another alternative is to compare mastectomy rates among screening participants and nonparticipants. However, as seen in the example of Elder et al.'s study [42], this is limited by uncertainty regarding the overdiagnosis rate, as well as issues of generalizability and healthy screener effects. A third alternative is to analyze the randomized trials [9], but these are decades old and out of date with recent screening practices and mastectomy use. Additionally, just as is the case for randomized trial-based estimates of overdiagnosis [11], randomized trials can overestimate overtreatment if follow-up is not long enough for the lead time or underestimate overtreatment if there is screening in the control group.

In summary, there are key limitations to all available methods for studying overtreatment of overdiagnosed cancer. The topic is fundamentally difficult to investigate, involving identifiability problems, data limitations, generalizability issues, and lack of recent randomized trials. However, the difficulty of studying overtreatment of overdiagnoses does not make it less important to study—the harm is already occurring and will not go away from being left unevaluated. In the current study, we developed an approach that has the advantage of being robust to data limitations (Figure 2), is generalizable because of the large and representative study cohort, and incorporates the uncertainty surrounding the extent of overdiagnosis. Our results demonstrate that the use of mastectomy could be commonplace for overdiagnosed patients and, consequently, that it is imprudent to ignore this medical harm. We hope this encourages other researchers to consider the issues of overdiagnosis when analyzing mastectomy use for early-stage breast cancer.

## 5. Conclusions

Of women diagnosed with breast cancer in 2013 at age ≥40 in the SEER 9 cancer registries, at most 18% underwent mastectomy for overdiagnosed cancer. This screening-associated overtreatment by mastectomy is less common than overdiagnosis itself but should not be assumed to be negligible. Because the US has a high rate of mastectomy, harms of breast cancer screening may be larger in the US than in many European nations.

## Figures and Tables

**Table 1:** Characteristics of breast cancer cases diagnosed at age ≥40 in 2013 and use of mastectomy.

**Characteristic**	**Cases**	**Treated with mastectomy (**%**)**	**Risk ratio (95**%** CI)**
Total	26017	8802 (33.8%)	

Stage, AJCC 7^th^			
0 (*in situ*)	5451	1439 (26.4%)	Ref.
I	10673	2988 (28.0%)	1.1 (1.0-1.1)
II	6353	2796 (44.0%)	1.7 (1.6-1.8)
III	1940	1306 (67.3%)	2.6 (2.4-2.7)
IV	1097	188 (17.1%)	0.6 (0.6-0.7)
NA	503	85 (16.9%)	0.6 (0.5-0.8)
Tumor size, cm			
0.0-0.9	5236	1271 (24.3%)	Ref.
1.0-1.9	8172	2407 (29.5%)	1.2 (1.1-1.3)
2.0-2.9	4308	1655 (38.4%)	1.6 (1.5-1.7)
3.0-3.9	2033	911 (44.8%)	1.8 (1.7-2.0)
4.0-4.9	1100	563 (51.2%)	2.1 (2.0-2.3)
5.0+	2043	1280 (62.7%)	2.6 (2.4-2.7)
NA	3125	715 (22.9%)	0.9 (0.9-1.0)
Regional lymph nodes positive			
0	14018	5177 (36.9%)	Ref.
1	2331	1043 (44.7%)	1.2 (1.2-1.3)
2+	2940	1760 (59.9%)	1.6 (1.6-1.7)
NA	6728	822 (12.2%)	0.3 (0.3-0.4)
Grade			
I	5556	1488 (26.8%)	Ref.
II	10580	3693 (34.9%)	1.3 (1.2-1.4)
III	7683	3122 (40.6%)	1.5 (1.4-1.6)
IV	161	59 (36.6%)	1.4 (1.1-1.7)
NA	2037	440 (21.6%)	0.8 (0.7-0.9)
Molecular status			
HER2− HR− (triple negative)	2064	830 (40.2%)	1.2 (1.1-1.2)
HER2− HR+	15049	5134 (34.1%)	Ref.
HER2+ HR−	867	395 (45.6%)	1.3 (1.2-1.4)
HER2+ HR+	1998	807 (40.4%)	1.2 (1.1-1.3)
NA	6039	1636 (27.1%)	0.8 (0.8-0.8)

Age, years			
40-49	4392	1956 (44.5%)	Ref.
50-64	10284	3519 (34.2%)	0.8 (0.7-0.8)
65-84	10062	3003 (29.8%)	0.7 (0.6-0.7)
85+	1279	324 (25.3%)	0.6 (0.5-0.6)
Race			
American Indian or Alaska Native	177	59 (33.3%)	1.0 (0.8-1.2)
Asian or Pacific Islander	2751	1024 (37.2%)	1.1 (1.1-1.2)
Black	2880	955 (33.2%)	1.0 (0.9-1)
White	20047	6724 (33.5%)	Ref.
Other and unknown	162	40 (24.7%)	0.7 (0.6-1)

NA, not available or not applicable; AJCC, American Joint Committee on Cancer; HR, hormone receptor status (negative if both estrogen and progesterone receptor status are negative, positive if either is positive); HER2, human epidermal growth factor receptor 2 neu status; CI, confidence interval; Ref., reference level.

Risk ratios are from univariate analyses and were estimated by unconditional maximum likelihood.

**Table 2:** Criteria used to rule out overdiagnosis.

**Cases are ruled out from being overdiagnosed if they show any of the following…**

(i) ≥2 invaded lymph nodes
(ii) ≥4 cm in diameter
(iii) Invasion to the pectoral fascia, muscle, or chest wall
(iv) Invasion to the skin of the breast with ulceration, or to the adjacent skin
(v) Distant metastasis

**Table 3:** Characteristics of the breast cancer cases not ruled out from being overdiagnoses.

**Characteristic**	**Cases**	**Treated with mastectomy (**%**)**	**Risk ratio (95**%** CI)**
Total	20229	5829 (28.8%)	

Stage, AJCC 7^th^			
0 (*in situ*)	4935	1154 (23.4%)	Ref.
I	10627	2966 (27.9%)	1.2 (1.1-1.3)
II	4232	1648 (38.9%)	1.7 (1.6-1.8)
III	0	-	-
IV	0	-	-
NA	426	61 (14.3%)	0.6 (0.5-0.8)
Tumor size, cm			
0.0-0.9	5131	1232 (24%)	Ref.
1.0-1.9	7573	2147 (28.4%)	1.2 (1.1-1.3)
2.0-2.9	3439	1253 (36.4%)	1.5 (1.4-1.6)
3.0-3.9	1416	586 (41.4%)	1.7 (1.6-1.9)
4.0-4.9	0	-	-
5.0+	0	-	-
NA	2661	611 (23.0%)	1.0 (0.9-1.0)
Regional lymph nodes positive			
0	12917	4422 (34.2%)	Ref.
1	1861	750 (40.3%)	1.2 (1.1-1.3)
2+	0	-	-
NA	5442	657 (12.1%)	0.4 (0.3-0.4)
Grade			
I	5035	1208 (24.0%)	Ref.
II	8301	2459 (29.6%)	1.2 (1.2-1.3)
III	5305	1826 (34.4%)	1.4 (1.3-1.5)
IV	116	34 (29.3%)	1.2 (0.9-1.6)
NA	1463	302 (20.6%)	0.9 (0.8-1.0)
Molecular status			
HER2− HR− (triple negative)	1398	485 (34.7%)	1.2 (1.1-1.3)
HER2− HR+	11726	3375 (28.8%)	Ref.
HER2+ HR−	521	204 (39.2%)	1.4 (1.2-1.5)
HER2+ HR+	1358	492 (36.2%)	1.3 (1.2-1.4)
NA	5217	1273 (24.4%)	0.8 (0.8-0.9)

Age, years			
40-49	3283	1264 (38.5%)	Ref.
50-64	7981	2330 (29.2%)	0.8 (0.7-0.8)
65-84	8057	2054 (25.5%)	0.7 (0.6-0.7)
85+	899	181 (20.1%)	0.5 (0.5-0.6)
Race			
American Indian or Alaska Native	138	41 (29.7%)	1.0 (0.8-1.4)
Asian or Pacific Islander	2174	719 (33.1%)	1.2 (1.1-1.2)
Black	2028	576 (28.4%)	1.0 (0.9-1.1)
White	15756	4472 (28.4%)	Ref.
Other and unknown	124	21 (16.9%)	0.6 (0.4-0.9)

NA, not available or not applicable; AJCC, American Joint Committee on Cancer; HR, hormone receptor status (negative if both estrogen and progesterone receptor status are negative, positive if either is positive); HER2, human epidermal growth factor receptor 2 neu status; CI, confidence interval; Ref., reference level.

Risk ratios are from univariate analyses and were estimated by unconditional maximum likelihood.

## Data Availability

All data used in the study analyses are publicly available from the Surveillance, Epidemiology, and End Results cancer registry program (https://seer.cancer.gov) of the US National Cancer Institute.

## Abbreviations

DCIS: Ductal Carcinoma in Situ;
OR: Odds Ratio;
SEER: Surveillance, Epidemiology, and End Results program.

## Supplementary Materials

Supplementary Materials Appendix S1: statistical supplement. Table S1: the 33 case characteristics used in our analysis of the proportions of cases treated with mastectomy. Table S2: the alternative sets of criteria that were used to rule out overdiagnosis in the sensitivity analyses. Figure S1: estimates of overtreatment by mastectomy using alternative criteria to rule out overdiagnosis. Figure S2: calibration of the model used to estimate probability of mastectomy. Figure S3: separate analysis of mastectomy use for overdiagnosed DCIS.
