# Supplementary material for: Use of Mastectomy for Overdiagnosed Breast Cancer in the United States: Analysis of the SEER 9 Cancer Registries
Source: J Cancer Epidemiol. 2019 Jan 22;2019:5072506. doi: 10.1155/2019/5072506 (PMC6362466; doi:10.1155/2019/5072506)
Supplement: Supplementary Materials — Appendix S1: statistical supplement. Table S1: the 33 case characteristics used in our analysis of the proportions of cases treated with mastectomy. Table S2: the alternative sets of criteria that were used to rule out overdiagnosis in the sensitivity analyses. Figure S1: estimates of overtreatment by mastectomy using alternative criteria to rule out overdiagnosis. Figure S2: calibration of the model used to estimate probability of mastectomy. Figure S3: separate analysis of mastectomy use for overdiagnosed DCIS. [file 5072506.f1.pdf]

# Appendices

*Use of Mastectomy for Overdiagnosed Breast Cancer*
*in the United States: Analysis of the SEER 9 Cancer Registries*

C. Harding, F. Pompei, D. Burmistrov, and R. Wilson

## Index

**Appendix S1:** Statistical supplement
**1** Method of obtaining bounds………………………………………………… 1
**2** Interpretation of the bounds - avoiding confusion……..…………………… 2
**3** Possibility of improving bounds…………………………………………….. 3
**4** Comparison with other statistical approaches………………………………. 3
**5** Validity of key assumption………………………………………………….. 4
**6** Sensitivity analysis for omitted variables…………………………………… 6
**7** References…………………………………………………………………… 9

**Table S1:** The 33 case characteristics used in our analysis of the proportions of cases treated with mastectomy……………………………………………………. 10

**Table S2:** The alternative sets of criteria that were used to rule out overdiagnosis in the sensitivity analyses……………………………………………………………… 12

**Figure S1:** Estimates of overtreatment by mastectomy using alternative criteria to rule out overdiagnosis.……………………………………..………………. 14

**Figure S2:** Calibration of the model used to estimate probability of mastectomy………………………………………………………………………………. 16

**Figure S3:** Separate analysis of mastectomy use for overdiagnosed DCIS…………... 17
.

# Appendix S1. Statistical Supplement

## 1 Method of obtaining bounds

This appendix provides additional detail on our method of obtaining bounds. As a reminder, we seek to obtain bounds on the proportion of breast cancer cases in our dataset that were overdiagnoses treated by mastectomy.

The initial step of our analysis was to exclude cases with features that rule out overdiagnosis. After making this exclusion, we are left with a group of cases, some of which were overdiagnosed and some of which were not. Below, our method of obtaining bounds is explained for this group in the large-sample limit.

Denote the recorded characteristics of breast cancer cases by $X$, and let the values of these characteristics for case $i$ be $x_i$. Further, let $M$ denote that mastectomy was performed and $\neg M$ that mastectomy was not performed. Let $V$ denote overdiagnosed cancer and $\neg V$ denote non-overdiagnosed (i.e., harmful) cancer. Suppose that the number of cases in our group is $n$ and the proportion of these cases that have been overdiagnosed is a known quantity, $r$.

We are interested in estimating the proportion of cases in the group that are overdiagnoses treated by mastectomy. In the large-sample limit, this is simply the probability of $M$ and $V$, $P(M, V)$, which we can expand as

$$\begin{aligned} P(M, V) &= \sum_{x_i} P(M, V \mid X = x_i) \cdot P(X = x_i) \\ &= \sum_{x_i} P(M \mid V, X = x_i) \cdot P(V \mid X = x_i) \cdot P(X = x_i), \end{aligned} \tag{1}$$

where the sum is over all unique $x_i$.

Currently, no one can identify cases that have been overdiagnosed. (Indeed, if overdiagnosed cases could be identified, they would not be treated, and there would be no need for our study.) For this reason, we make the following assumption: Conditional on the recorded characteristics of a case at diagnosis, the probability of mastectomy would not be different if the case was overdiagnosed cancer or if it was non-overdiagnosed cancer. In other words, conditional on case characteristics, the counterfactual probability of treatment by mastectomy is the same for overdiagnosed and non-overdiagnosed cancer. So

$$P(M \mid V, X = x_i) = P(M \mid \neg V, X = x_i) = P(M \mid X = x_i)$$

We will return to this key assumption in more detail soon. For now, substituting this into Eq. 1, we have

$$P(M, V) = \sum_{x_i} P(M \mid X = x_i) \cdot P(V \mid X = x_i) \cdot P(X = x_i) \tag{2}$$

On the right hand side, $P(M \mid X = x_i)$ is the probability of mastectomy conditional on recorded characteristics, and can be estimated directly from the data (for example using a regression model, as explained in the Methods). Additionally, $P(X = x_i)$ is the probability that a case has characteristics $x_i$, and can be estimated as the observed probability that $X = x_i$ in the data,

$$\hat{P}(X = x_i) = \frac{1}{n} \sum_{j=1}^{n} \mathbf{1}\left[x_j = x_i\right],$$

where $\mathbf{1}$ is the indicator function. Only $P(V \mid X = x_i)$, the probability of overdiagnosis conditional on the observed characteristics, is unknown.

We have assumed that the proportion of the $n$ cases that are overdiagnosed is a known quantity $r$. This restricts the values that $P(V \mid X = x_i)$ can take, since $r = \sum_{x_i} P(V \mid X = x_i) \cdot P(X = x_i)$. By distributing the allowed values of $P(V \mid X = x_i)$ in such a way that maximizes the value of Eq. 2, we obtain an upper bound on the amount of overtreatment by mastectomy after overdiagnosis. Similarly, by distributing the allowed values of $P(V \mid X = x_i)$ to minimize Eq. 2, we obtain a lower bound.

The upper bound is obtained by assigning a value of 1 to $P(V \mid X = x_i)$ for the proportion $r$ of cases in our dataset for which $P(M \mid X = x_i)$ is largest, and assigning a value of 0 otherwise. Similarly, the lower bound is obtained by assigning 1 to $P(V \mid X = x_i)$ for the proportion $r$ of cases for which $P(M \mid X = x_i)$ is smallest, and assigning 0 otherwise.

In this way, we obtain bounds on the proportion of breast cancers cases in the study population that were overdiagnosed and overtreated by mastectomy. This provides a full account of the methods used to obtain bounds, but several topics should be addressed in more detail: First, how the bounds should be interpreted. Second, whether it is possible to improve the bounds. Third, how our methods compare with other statistical approaches. Fourth, the validity of the key assumption. Fifth, the possible effects of omitted variables. These are discussed in the following sections.

## 2 Interpretation of the bounds - avoiding confusion

The bounds apply to the proportion of cases that were overdiagnosed and treated by mastectomy in the study cohort. Note that the bounds do not apply to the probability of overtreatment by mastectomy for individual cases: for any individual case, the probability of overtreatment by mastectomy could be much lower or higher than the bounds.

Additionally, the bounds do not provide any information about the location of the true value within the bound. They only demark the range of values that is possible. For example, if the analysis shows 3%-15% of cases are mastectomy-treated overdiagnoses, then this does not provide any information about whether the true value is near the middle of this range, 9%, or nearer the edges.

## 3 Possibility of improving bounds

Improvements to the bounds might be achieved if it is reasonable to exclude more cases from being overdiagnosed based on their observed features. This is explored in Table S2 and Figure S2 of Appendix S2, but only has small effects on the bounds.

The bounds might also be improved by placing further restrictions on $P(V \mid X = x_i)$. In the prior literature, it is commonly assumed that the percentage of overdiagnosed patients is higher among *in situ* cases than it is among invasive cases. It is also reasonable to assume that the percentage of overdiagnosed patients is higher for cases with no positive lymph nodes than it is for cases with positive lymph nodes; as discussed in the Methods of the main text, it is likely that cases with positive lymph nodes can only be overdiagnoses if the lymph node findings are false positives. Both of these restrictions on $P(V \mid X = x_i)$ were explored, but we found that they did not change the resulting bounds substantially, so we have not applied them for the results presented in this study.

One might also obtain tighter bounds by assuming that the proportion of overdiagnosed cases treated by mastectomy cannot be greater than the proportion of all cases treated by mastectomy. However, we investigated this assumption and found that it is not as reasonable as it first appears because rates of mastectomy are often higher for early-stage breast cancers, some of which are likely overdiagnosed, than for more advanced breast cancers, almost none of which are overdiagnosed.

## 4 Comparison with other statistical approaches

### 4.1 Propensity scores

Suppose that we want to study the effect of an intervention on an outcome in a group of individuals. For individual $i$, the propensity score is the probability that $i$ is assigned to receive the intervention, conditional on the characteristics that have been recorded for $i$ (2). If the recorded characteristics block all open back-door paths from the intervention to the outcome, then controlling for the propensity score prevents confounding when evaluating the effect of the intervention on the outcome (3).

In our case, the intervention is being overdiagnosed with cancer (vs. being correctly diagnosed with cancer) and the outcome is use of mastectomy. The intervention is unobservable, a feature of our analysis that makes it different from previous work and makes propensity scores inapplicable. To address this, we predict the outcome based on variables that block all open paths from the intervention to the outcome, including both open back door paths and open forward paths (see Figure 1 in this appendix). Then we obtain an upper bound on the effects of the intervention by assuming the cases with the largest predicted values belonged to the intervention group. Similarly, we obtain a lower bound by assuming cases with the least predicted values belonged to the intervention group.

## 4.2 Regression standardization

Our approach to obtaining bounds can also be compared with regression standardization (1) (pp. 386-388). In regression standardization, we observe some outcome of interest $Y$, as well as case characteristics $X$, and a variable $Z$. For some group of patients, we wish to estimate how outcome Y would differ if the patients in this group had a certain value of $Z = z'$, instead of the values of Z they actually have. For example, Y might be mortality related to some kind of infection, Z might be the type of medicine a patient receives, and we might be interested in estimating what mortality would be if all patients in a particular subgroup had received the specific medicine $z'$ instead of whatever medicines they actually received. Under the assumption that characteristics $X$ are sufficient for confounding control and some additional conditions (1), we can estimate this value as

$$\sum_{x_i} E(Y \mid Z = z', X = x_i) \cdot P(X = x_i),$$

where $E(Y \mid Z = z', X = x_i)$ is estimated using a regression model and the weights $P(X = x_i)$ are the probabilities that patients in the subgroup of interest have characteristics $X = x_i$.

In the present paper, we wish to calculate a somewhat different quantity, Eq. 2, which we can rewrite as $\sum_{x_i} E(M \mid X = x_i) \cdot P(V \mid X = x_i) \cdot P(X = x_i)$. Similar to regression standardization, we use a regression model to estimate $E(M \mid X = x_i)$, and the weights $P(X = x_i)$ are estimated directly from the data. However, we have the disadvantage that we also need a second set of weights, $P(V \mid X = x_i)$, which are unobservable. So, we proceed by trying the best and worst possible values of $P(V \mid X = x_i)$, and thereby obtain bounds on Eq. 2.

# 5 Validity of key assumption

The key assumption in our analysis is that, conditional on observed characteristics at diagnosis, the probability of mastectomy does not differ between overdiagnosed and non-overdiagnosed cases. An important limitation to our work, therefore, is that there could still be relevant characteristics that we have omitted when estimating $P(M \mid X = x_i)$ because they are not available in the data source.

Figure 1 presents a causal diagram of our analysis (1), which shows the types of omitted variables that do and do not need to be included in our regression-based predictions of mastectomy, in order to obtain unbiased estimates of the proportion of cases that are overdiagnoses treated by mastectomy.

A concrete example of omitted variables that would need to be included is as follows: We have no information about personal preferences and motivations, and this information is likely to be absent from most data sources. Women who are especially vigilant about their breast cancer risk may participate in screenings more frequently than average, and therefore be especially likely to be overdiagnosed. Because of their caution about the risks of breast cancer, these women may also be especially likely to select mastectomy as treatment, relative to others whose cases have the same recorded characteristics at diagnosis. In this hypothetical example, lacking information on personal preferences

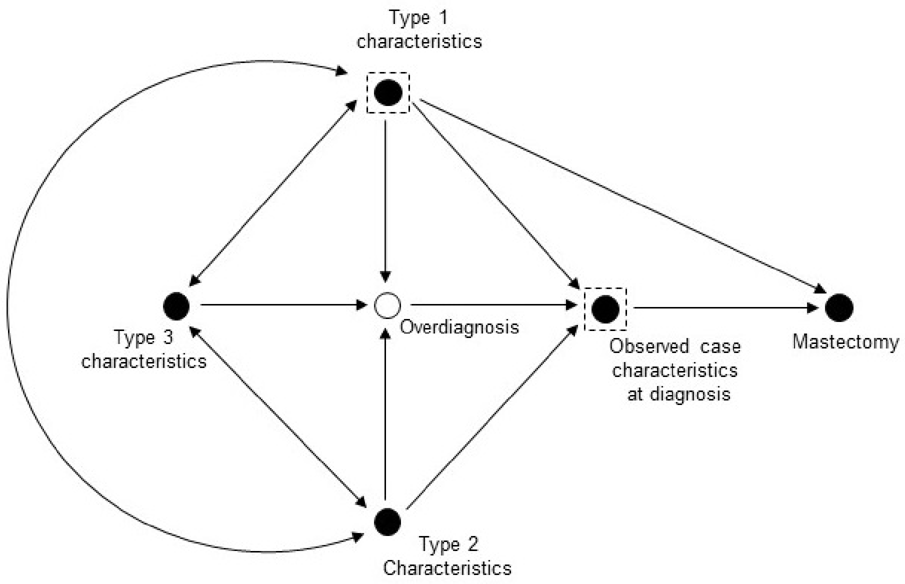


Figure 1: Causal diagram showing the variables relevant to the analysis. Mastectomy and overdiagnosis are binary variables. Overdiagnosis is unobservable. Type 1, 2, and 3 characteristics differ in the causal relationships that they have with observed characteristics at diagnosis and mastectomy. Type 1 characteristics are parents of both observed characteristics at diagnosis and mastectomy, while type 2 characteristics are parents of observed characteristics at diagnosis, but not mastectomy, and type 3 characteristics are parents of neither. The variable "mode of detection" (screening vs. symptomatic) is likely to be a type 2 characteristics, or a type 1 characteristics with a weak direct effect on mastectomy. Variables surrounded by dashed boxes need to be included in the regression-based predictions of mastectomy probabilities, in order to block all open paths* from overdiagnosis to mastectomy and thereby obtain correct bounds on the proportion of cases that are overdiagnoses treated by mastectomy. *Including open forward and open back-door paths.

and motivations would be equivalent to omitting a type 1 characteristics (see Fig. 1), and could lead us to underestimate the amount of overtreatment by mastectomy that follows overdiagnosis. On the other hand, it is also possible that women with family histories of breast cancer may be especially likely to be diagnosed with harmful breast cancers, rather than overdiagnosed breast cancers, and may also be especially likely to select mastectomy. In this hypothetical example, lacking information on personal preferences and motivations would again be equivalent to omitting a type 1 characteristic, but might lead us to overestimate overtreatment by mastectomy.

The assumption of no omitted variables used in our methods corresponds to "no omitted variables," "no unobserved confounders," and related assumptions that are used in observational studies so routinely that they often go unmentioned. As is true for any observational study, even if the assumption were satisfied, we could never be certain that was the case. However, in the following section, we describe an analysis that was used to investigate the sensitivity of our findings to violations of the assumption.

## 6 Sensitivity analysis for omitted variables

This section describes the methods that we used to investigate the sensitivity of our main results to omitted variables and missing values. Recall that our main analysis relies on predictions of mastectomy obtained from a regression model. The sensitivity analysis assumes that the predictions of mastectomy are not systematically biased, but that omitted variables and missing values could produce unmodelled variance around the predicted values. The consequences of unmodelled variance of different sizes are evaluated.

### 6.1 Background

Suppose that our analysis includes $n$ women who have been diagnosed with breast cancer. For each of these women $i$, let $q_i$ be the probability she receives mastectomy conditional on all observable characteristics associated with her and her case. Because some observable characteristics are not recorded in our data source (or any data source), we cannot obtain $q_i$ itself. However, we have an estimate of it from our regression analysis, which predicts receipt of mastectomy based on the characteristics recorded in the data source. Let our estimate of $q_i$ be called $p_i$.

In practice, $p_i$ may not be an especially good estimate of $q_i$, particularly because we do not have information on all characteristics that are relevant to receipt of mastectomy. The potential inaccuracy of $p_i$ concerns us because the values $q_i$ are required by our method of obtaining bounds on the rate of overdiagnoses treated by mastectomy.

### 6.2 Assumptions

Despite the inaccuracy of $p_i$, we are willing to make some assumptions that bound the differences between $p_i$ and $q_i$.

Consider any subgroup of individuals $S$ who share the same recorded characteristics (for $j, k \in S$, $x_j = x_k$) and therefore also have the same regression-based estimator of mastectomy probability, $\hat{p}_j$. We assume that the expected

value of this estimator, $E(\hat{p}_j \mid j : j \in S)$, equals the expected value of the actual probability of mastectomy in the subgroup, $E(q_j \mid j : j \in S)$. In words, we are assuming that our mastectomy predictions are not systematically biased. Another way to look at this is that we are assuming the regression estimator of $q_i$ is well calibrated. (For our analysis, calibration results are presented in Appendix S2, Figure S2 and show generally good performance.)

Additionally, we assume that the actual odds of mastectomy and the estimated odds can differ by a by no more than some factor $\phi$. In other words, we assume

$$\frac{p_i}{\phi \cdot (1 - p_i)} \leq \frac{q_i}{1 - q_i} \leq \frac{\phi \cdot p_i}{1 - p_i}. \tag{3}$$

The factor $\phi$ controls the amount of unmodelled variation that we are evaluating in the sensitivity analysis.

## 6.3 Procedure

Using these two assumptions, we can conduct a sensitivity analysis as follows: Let $y_i$ be the odds for probability $p_i$. That is, $y_i = p_i/(1 - p_i)$. Define the lower and upper odds $y'_i = y_i/\phi$ and $y''_i = \phi \cdot y_i$, and decompose $p_i$ as

$$p_i = w \cdot p'_i + (1 - w) \cdot p''_i,$$

where $w$ is a weight, $y'_i = p'_i/(1 - p'_i)$, and $y''_i = p''_i/(1 - p''_i)$.

Solving for $w$, $p'_i$, and $p''_i$ gives

$$w = \frac{p_i + \phi - p_i \cdot \phi}{\phi + 1}$$

$$p'_i = \frac{p_i}{p_i + \phi - p_i \cdot \phi}$$

$$p''_i = \frac{p_i \cdot \phi}{p_i \cdot \phi - p_i + 1}$$

Next, we create a new, artificial dataset of patients. For each patient $i$ in our original dataset, we include two artificial patients in the new dataset, $i'$ and $i''$, with estimated probabilities of mastectomy $p'_i$ and $p''_i$ and weights $w$ and $1 - w$, respectively.

As explained in Section 1 of this document, our method of obtaining upper bounds consists of summing the largest $\lceil n \cdot r \rceil$ of the $q_i$ values, where $n$ is the total number of included cases and $r$ is the proportion of included cases that are overdiagnosed. For the sensitivity analysis, we instead use the artificial dataset and sum the $\lceil m \rceil$ largest of the included probabilities of mastectomy, where $m$ is chosen such that the sum of the associated weights in the artificial dataset equals $n \cdot r$. The analysis proceeds analogously for lower bounds.

## 6.4 Correctness

We argue that this process results in the largest possible bound that can be obtained without exceeding the $\phi$ value that we have chosen. If our sum includes only double-primed entries ($p''_i$, $p''_j$, $p''_k$,...), then we are done because the sum cannot be increased without exceeding $\phi$. However, if the sum includes any single-primed entries ($p'_i$, $p'_j$, $p'_k$,...), then we are left with an unresolved

question: Could we have increased the value of our sum by, for some $i$, splitting $i$ differently than into $i'$ and $i''$, while still obeying our assumptions?

This is not possible for the following reason: If $i'$ is in our sum, then $i''$ must be too because $p'_i < p''_i$. Additionally, if both $i'$ and $i''$ are in our sum, then we are including the entirety of patient $i$ in the sum (included weight = 1). If all of patient $i$ is included in the sum, then the unbiasedness assumption implies that the contribution to the sum is the same regardless of how we choose to split $i$. Therefore, the sum cannot be increased further without exceeding the parameter $\phi$.

## 6.5 Choice of $\phi$

The usefulness of the sensitivity analysis depends on investigating values of $\phi$ that that are likely to account for plausible levels of omitted variables bias. $\phi$ is defined such that, for any case, the actual odds of mastectomy should not be more than $\phi$-fold greater than the estimated odds of mastectomy and — additionally — the actual odds of mastectomy should not be less than $1/\phi$-fold smaller than the estimated odds of mastectomy (Equation 3). This means that, for any group of cases with the same recorded characteristics, the odds ratio comparing the lowest and highest actual odds of mastectomy in the group should be at most $\phi^2$.

Accordingly, reasonable values of $\phi$ can be chosen by considering the odds ratios for mastectomy that are observed in analyses of the recorded variables. For example, among the recorded variables in Table 3, the largest odds ratio was 4.9, which suggests that using a $\phi^2$ value of 4.9 — and an associated $\phi$ value of 2.2 — is equivalent to testing the sensitivity of the analysis to omission of a major variable.

Because of the relationship between $\phi^2$ and the odds ratios of omitted variables, we refer to $\phi^2$ as the "sensitivity parameter" and the "omitted variables' odds ratio for mastectomy" in the main text.

## 6.6 Relationship with previous work

Our sensitivity analysis is similar to methods that have previously been used to assess the sensitivity of many propensity score-based studies to hidden bias (3-4). In these studies, researchers generally use recorded characteristics to estimate the probability of treatment as a means of evaluating causal effects. However, the researchers are concerned that estimated probabilities of treatment may differ from actual probabilities of treatment due to the presence of hidden or omitted variables. Sensitivity analyses are then undertaken, supposing the existence of a hidden variable that is associated with treatment with a known odds ratio.

## 6.7 Results of the sensitivity analysis

As described in the main text, applying the sensitivity analysis with sensitivity parameters ranging from odds ratio = 1 to odds ratio = 25 ($\phi^2 = 1$ to $\phi^2 = 25$) showed that our results were largely robust to bias from omitted variables and missing values.

## 7 References

1. Rothman KJ, Greenland S, Lash T. Modern epidemiology. 3rd ed. Philadelphia, PA: Lippincott Williams & Wilkins; 2008. pp 183-209, 386-388.

2. Rosenbaum, P. Observational studies. 2nd ed. New York, NY: Springer-Verlag; 2002. Ch. 4.

3. Morgan SL, Winship C. Counterfactuals and causal inference. Cambridge University Press; 2015.

4. McCaffrey DF, Ridgeway G, Morral AR. Propensity score estimation with boosted regression for evaluating causal effects in observational studies. Psychological methods. 2004;9(4):403.

**Table S1:** The 33 case characteristics used in our analysis of the proportions of cases treated with mastectomy.

*Patient characteristics*

- Age at diagnosis (in years)
- Marital status (divorced, married, separated, single, unknown, unmarried or domestic partner, widowed)
- Insurance recode (any Medicaid, unknown, insured, insured/no specifics, uninsured)
- Race (American Indian and Alaska Native, Asian or Pacific Islander, black, white, unknown)
- Spanish-Hispanic-Latino (yes or no)
- Race-ethnicity (multiple categories)
- Number of prior tumors recorded in SEER

*Disease characteristics*

- Tumor size (in millimeters)
- Tumor size (various categories)
- Tumor extension (multiple categories)
- Lymph node status (multiple categories)
- Metastases at diagnosis (multiple categories)
- Estrogen receptor status (positive, negative, borderline, unknown)
- Progesterone receptor status (positive, negative, borderline, or unknown)
- Human epidermal growth factor 2 neu (HER2) status (positive, negative, borderline, or unknown)

- Molecular subtype (HR- HER2- [triple negative], HR- HER2+, HR+ HER2-, HR+ HER2+, unknown)
- Regional lymph nodes positive (count)
- American Joint Committee on Cancer (AJCC) 7$^{th}$-ed. stage
- AJCC 7$^{th}$ ed. T stage
- AJCC 7$^{th}$-ed. M stage
- AJCC 7$^{th}$-ed. N stage
- Histology broad groupings (many categories)
- Histologic type ICD-O-3 (many categories)
- Laterality (bilateral, left, right, only one side by side unknown, unknown)
- Grade (I, II, III, IV, unknown),
- Primary site in breast (several categories, including quadrant information)

*Regional characteristics*

- SEER registry
- State and county
- Percent of county population living in an urban area in 2010
- Rural-urban continuum code
- Percent of county population living below poverty in 2009-2013
- Percent of county population living below 150% of poverty in 2009-2013
- Percent of county population living below 200% of poverty in 2009-2013.

**Table S2:** The alternative sets of criteria that were used to rule out overdiagnosis in the sensitivity analyses. Criteria 1 are the same as those used in the main paper, while Criteria S1 and S2 progressively exclude more cases from being overdiagnoses. Differences in the criteria are highlighted in red.

| **Criteria Set** | **Cases are ruled out from being overdiagnosed if …** |
|---|---|
| Main paper | • There are ≥2 invaded lymph nodes<br>• The tumor is ≥4 cm in diameter<br>• There is invasion to the pectoral facia, muscle, or chest wall<br>• There is invasion to the skin of the breast with ulceration, or to the adjacent skin<br>• There is distant metastasis |
| Criteria S1 | • There is ≥**1** invaded lymph node<br>• The tumor is ≥4 cm in diameter<br>• There is invasion to the pectoral facia, muscle, or chest wall<br>• There is invasion to the skin of the breast with ulceration, or to the adjacent skin<br>• There is distant metastasis |
| Criteria S2 | • There is ≥**1** invaded lymph node<br>• The tumor is ≥**3** cm in diameter |

| | |
|---|---|
| | • There is invasion to the pectoral facia, muscle, or chest wall<br>• There is invasion to the skin of the breast with ulceration, or to the adjacent skin<br>• There is distant metastasis |

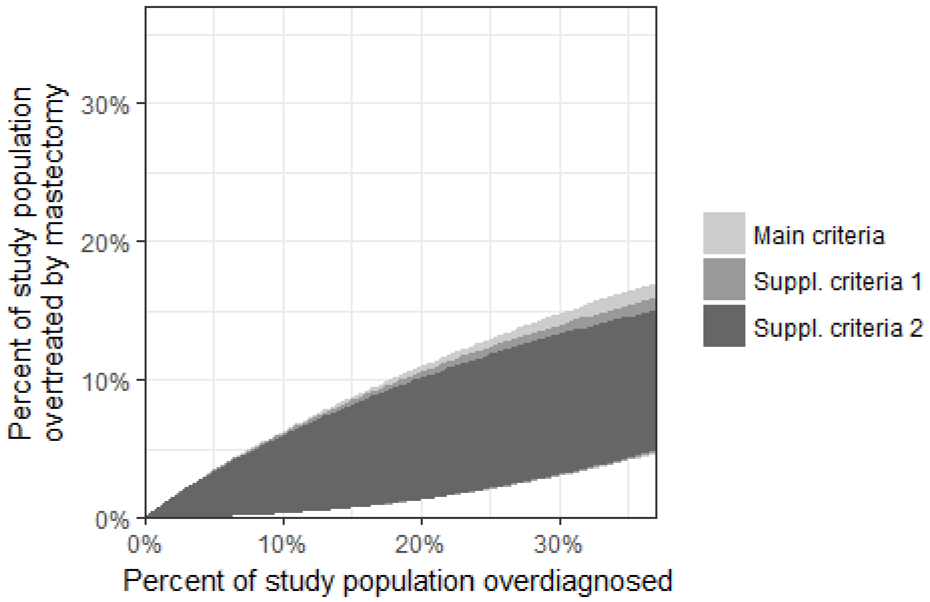


**Figure S1:** Supplementary analyses of overtreatment by mastectomy in the study population: women diagnosed with breast cancer at age ≥40 in 2013 in the SEER 9 cancer registries

In this supplementary analysis, we investigated 3 different criteria for ruling out overdiagnosis. Overall, the analysis shows that similar bounds are obtained on the rate of mastectomy-treated overdiagnosis, regardless of the criteria used.

For each set of criteria, the shaded region shows the minimum and maximum possible percentages of the study population who were overdiagnosed and overtreated by mastectomy. To obtain the minimum or maximum possible rate of mastectomy-treated overdiagnoses, it is necessary to assume that a certain proportion of the study population is being overdiagnosed (x-axis). Results for the criteria used in the main paper are shown in light gray. These are the least exclusive criteria. Results for Criteria S1 (like the main criteria, but also excluding cases with 1 invaded node) are shown in medium gray. Results for Criteria S2 (like Criteria S1, but also excluding cases 3-4 cm in size) are shown in dark gray. Lower bounds on overtreatment by mastectomy are almost entirely the same for all sets of criteria. Upper bounds are generally similar for all sets of criteria, but vary by a few percentage points at high rates of overdiagnosis.

The table below shows the number of cases that remain (e.g., that are not ruled out from being overdiagnoses) after applying each set of criteria. Also shown is the number and percent of these cases that were treated by mastectomy.

| | **Total cases** | **Cases treated with mastectomy (%)** |
|---|---|---|
| Using main criteria | 20220 | 5829 (28.8%) |
| Using suppl. criteria 1 | 18346 | 5072 (27.6%) |
| Using suppl. criteria 2 | 17168 | 4598 (26.8%) |

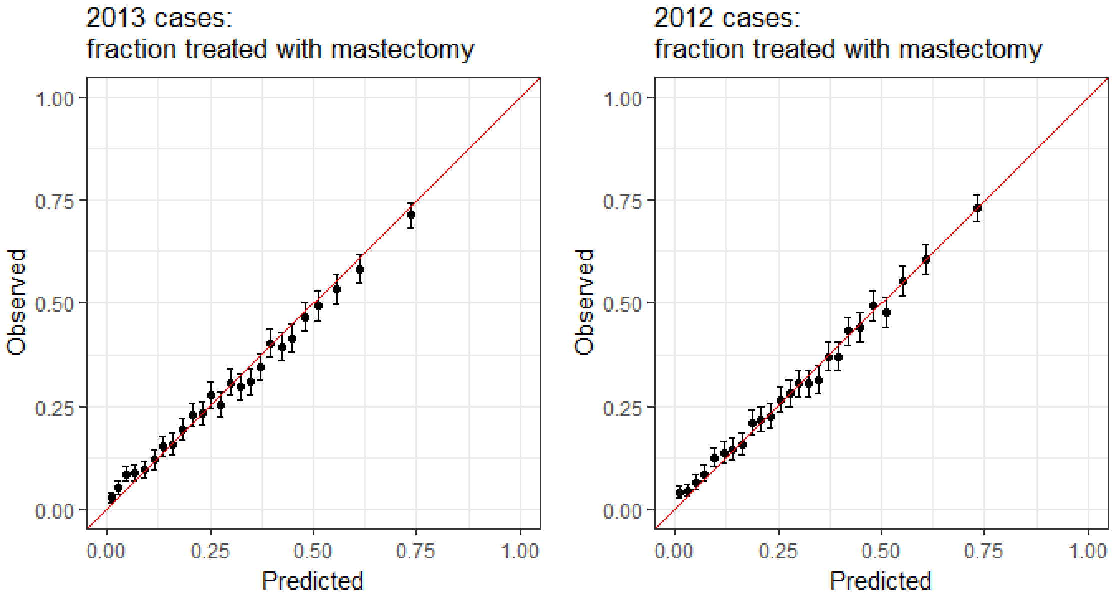


**Figure S2:** Calibration of the estimated probabilities of mastectomy

Based on the characteristics of breast cancer diagnoses in 2013, the probabilities of treatment by mastectomy were estimated using a random forest classification model. The data were ordered from lowest to highest probability of mastectomy and split into 25 equal-sized groups to assess calibration. In the left plot, the predicted and observed probabilities are compared for 2013, showing good calibration with slight deviation at the lowest values. In the right plot, the comparison is repeated for test set data—cases diagnosed in 2012—using predictions from the random forest that was trained to cases diagnosed in 2013. Good calibration is seen again. Confidence intervals are 95% binomial.

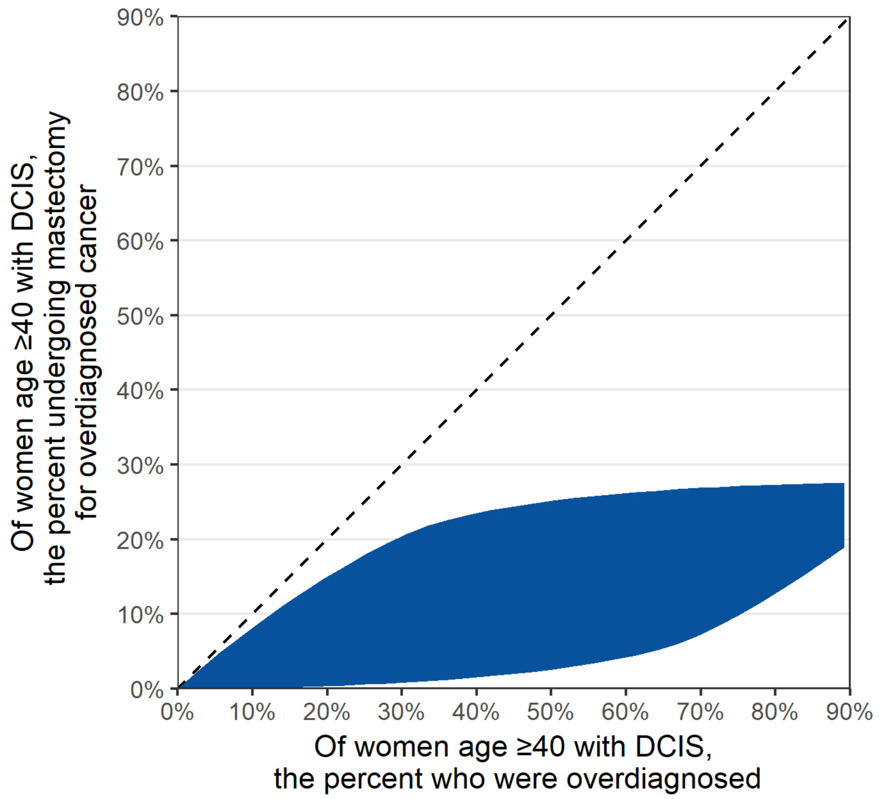


**Figure S3:** Separate Analysis of Mastectomy Use for Overdiagnosed DCIS

In this supplement, we performed a separate analysis of use of mastectomy as a treatment for overdiagnosed ductal carcinoma *in situ* (DCIS). The analysis is limited to women who were diagnosed with DCIS at age ≥40, during the year 2013, and in the SEER 9 cancer registries. For each overdiagnosis percentage shown on the x-axis, the shaded region demarks the range of possible percentages of overtreatment by mastectomy (y-axis) that are consistent with the known characteristics of the DCIS cases in the study. For example, if the overdiagnosis percentage was 75% then, based on the known characteristics of the DCIS cases, we can conclude that the percentage of overtreatment by mastectomy was somewhere between 9% and 27%.

Figure 1 of the main article shows a similar analysis that includes both *in situ* and invasive breast cancers.
